# Supplementary material for: Unveiling the Texture Secrets of Morchella Germplasm: Advanced Grading and Quality Assessment Through Texture Profile Analysis (TPA)
Source: Foods. 2025 Jan 1;14(1):87. doi: 10.3390/foods14010087 (PMC11719484; doi:10.3390/foods14010087)
Supplement: Supplementary file 1 [file foods-14-00087-s001.zip › foods-3375746-Supplementary tables.pdf]

Table S1. Normality test of texture parameters of *Morchella* pileus.

Strain species	Characteristic	Range			K-S value	Sig value	Skewness	Kurtosis
		Absolute value	Positive	Negative				
M.importuna	Hardness	0.089	0.089	-0.056	0.089	0.000	0.908	1.402
	Springiness	0.036	0.036	-0.019	0.036	0.200	0.239	0.142
	Cohesiveness	0.061	0.061	-0.022	0.061	0.015	0.299	-0.349
	Gumminess	0.091	0.091	-0.065	0.091	0.000	1.091	1.437
	Chewiness	0.093	0.093	-0.081	0.093	0.000	1.310	2.177
	Resilience	0.074	0.074	-0.053	0.074	0.001	0.826	0.584
M.sextelata	Hardness	0.051	0.051	-0.035	0.051	0.010	0.425	-0.339
	Springiness	0.040	0.039	-0.040	0.040	0.124	0.012	-0.619
	Cohesiveness	0.059	0.059	-0.029	0.059	0.002	0.384	-0.101
	Gumminess	0.094	0.094	-0.062	0.094	0.000	0.827	0.284
	Chewiness	0.117	0.117	-0.099	0.117	0.000	1.031	0.522
	Resilience	0.102	0.102	-0.066	0.102	0.000	0.931	0.691
M.eximia	Hardness	0.083	0.083	-0.046	0.083	0.000	0.750	0.351
	Springiness	0.042	0.042	-0.032	0.042	0.200	0.047	-0.077
	Cohesiveness	0.047	0.047	-0.027	0.047	0.200	0.335	-0.200
	Gumminess	0.093	0.093	-0.053	0.093	0.000	0.903	0.627
	Chewiness	0.111	0.111	-0.064	0.111	0.000	0.957	0.575
	Resilience	0.114	0.114	-0.073	0.114	0.000	0.998	0.533

Table S2. Normality test of texture parameters of *Morchella* stipe.

Strain species	Characteristic	Range			K-S value	Sig value	Skewness	Kurtosis
		Absolute value	Positive	Negative				
M.importuna	Hardness	0.114	0.114	-0.070	0.114	0.000	1.093	1.220
	Springiness	0.038	0.024	-0.038	0.038	0.200	-0.189	0.032
	Cohesiveness	0.033	0.018	-0.033	0.033	0.200	-0.029	-0.135
	Gumminess	0.079	0.079	-0.037	0.079	0.000	0.692	0.537
	Chewiness	0.066	0.066	-0.032	0.066	0.005	0.841	1.466
	Resilience	0.051	0.051	-0.033	0.051	0.074	0.388	-0.008
M.sextelata	Hardness	0.122	0.122	-0.073	0.122	0.000	1.316	2.152
	Springiness	0.037	0.025	-0.037	0.037	0.185	-0.306	0.172
	Cohesiveness	0.030	0.030	-0.025	0.030	0.200	0.132	-0.085
	Gumminess	0.088	0.088	-0.061	0.088	0.000	1.100	1.580
	Chewiness	0.096	0.096	-0.058	0.096	0.000	0.997	1.085
	Resilience	0.043	0.043	-0.033	0.043	0.059	0.223	0.265
M.eximia	Hardness	0.102	0.102	-0.057	0.102	0.000	0.811	-0.006
	Springiness	0.038	0.033	-0.038	0.038	0.200	-0.052	0.072
	Cohesiveness	0.059	0.059	-0.027	0.059	0.039	0.198	-0.407
	Gumminess	0.082	0.082	-0.047	0.082	0.000	0.671	-0.106
	Chewiness	0.082	0.082	-0.054	0.082	0.000	0.961	0.864
	Resilience	0.075	0.075	-0.050	0.075	0.002	1.288	3.865

Table S3. Comprehensive evaluation of texture parameters of *Morchella* pileus.

Strain	*F* value	Ranking	Strain	*F* value	Ranking	Strain	*F* value	Ranking	Strain	*F* value	Ranking
M1	0.637	124	M55	0.449	178	M109	0.422	186	M163	1.111	15
M2	0.593	141	M56	0.875	64	M110	0.479	168	M164	0.783	82
M3	1.061	24	M57	0.986	37	M111	0.331	202	M165	0.640	123
M4	1.270	8	M58	0.931	48	M112	0.448	179	M166	0.673	111
M5	0.731	93	M59	0.670	113	M113	0.605	138	M167	0.868	66
M6	1.044	28	M60	0.504	162	M114	0.531	155	M168	0.705	104
M7	0.945	45	M61	0.874	65	M115	0.494	164	M169	0.705	105
M8	0.840	74	M62	0.720	96	M116	0.359	199	M170	0.982	40
M9	1.134	12	M63	0.686	107	M117	0.636	125	M171	0.673	112
M10	0.818	78	M64	0.435	183	M118	0.348	200	M172	1.309	5
M11	0.563	147	M65	0.454	175	M119	0.124	213	M173	0.430	185
M12	0.509	160	M66	0.553	152	M120	0.395	192	M174	0.660	116
M13	1.048	25	M67	0.418	190	M121	0.154	212	M175	0.862	68
M14	1.384	4	M68	0.528	157	M122	0.271	206	M176	0.841	73

M15	1.419	3	M69	0.743	91	M123	0.979	41	M177	1.025	29
M16	0.943	46	M70	0.242	208	M124	1.014	31	M178	0.985	39
M17	0.389	193	M71	1.003	33	M125	0.273	205	M179	0.952	44
M18	0.674	110	M72	0.720	95	M126	0.415	191	M180	0.775	87
M19	0.714	99	M73	1.198	9	M127	0.708	101	M181	0.985	38
M20	0.608	137	M74	0.627	128	M128	0.456	174	M182	0.739	92
M21	0.470	170	M75	0.190	209	M129	0.263	207	M183	0.808	80
M22	0.620	132	M76	0.582	143	M130	1.046	27	M184	1.092	20
M23	0.969	42	M77	0.461	173	M131	1.100	18	M185	0.712	100
M24	1.275	7	M78	0.614	135	M132	0.783	83	M186	0.839	75
M25	0.910	51	M79	0.882	59	M133	0.836	76	M187	0.908	52
M26	0.502	163	M80	0.624	131	M134	0.889	56	M188	1.091	21
M27	0.714	98	M81	0.526	159	M135	1.000	36	M189	0.506	161
M28	0.884	58	M82	0.626	129	M136	0.453	176	M190	0.419	188
M29	0.783	84	M83	0.826	77	M137	0.422	187	M191	1.276	6
M30	0.441	180	M84	0.625	130	M138	0.897	55	M192	1.458	2
M31	0.882	60	M85	0.166	210	M139	0.604	139	M193	1.132	13
M32	1.005	32	M86	0.309	204	M140	0.311	203	M194	0.657	117
M33	0.875	63	M87	0.434	184	M141	0.450	177	M195	0.879	62
M34	0.643	122	M88	0.608	136	M142	0.164	211	M196	0.670	114
M35	0.706	103	M89	0.779	85	M143	0.661	115	M197	0.572	145
M36	0.620	133	M90	0.470	171	M144	0.685	108	M198	0.897	54
M37	0.656	118	M91	0.539	154	M145	0.598	140	M199	1.002	34
M38	0.914	49	M92	0.489	165	M146	0.651	121	M200	0.776	86
M39	0.435	182	M93	0.558	151	M147	0.676	109	M201	0.629	127
M40	0.440	181	M94	0.364	198	M148	0.962	43	M202	0.562	149
M41	1.101	17	M95	0.558	150	M149	0.881	61	M203	0.748	90
M42	0.897	53	M96	0.380	196	M150	0.526	158	M204	1.152	11
M43	1.160	10	M97	0.575	144	M151	1.118	14	M205	0.810	79
M44	0.724	94	M98	0.617	134	M152	0.886	57	M206	0.808	81
M45	0.484	167	M99	1.002	35	M153	0.347	201	M207	0.760	89
M46	0.488	166	M100	0.864	67	M154	1.100	19	M208	1.108	16
M47	0.376	197	M101	0.044	214	M155	0.858	69	M209	1.630	1
M48	0.466	172	M102	0.706	102	M156	0.383	194	M210	0.419	189
M49	0.850	71	M103	1.048	26	M157	0.478	169	M211	0.853	70
M50	0.656	119	M104	0.719	97	M158	0.541	153	M212	0.585	142
M51	1.079	22	M105	0.562	148	M159	0.630	126	M213	0.566	146
M52	0.910	50	M106	0.383	195	M160	0.528	156	M214	0.655	120
M53	0.696	106	M107	0.848	72	M161	1.072	23			
M54	0.762	88	M108	0.931	47	M162	1.018	30			

Table S4. Comprehensive evaluation of texture parameters of *Morchella* stipe.

Strain	*F* value	Ranking	Strain	*F* value	Ranking	Strain	*F* value	Ranking	Strain	*F* value	Ranking
M1	0.576	27	M55	0.904	6	M109	0.320	95	M163	0.437	46
M2	0.200	148	M56	0.023	200	M110	-0.010	205	M164	0.291	107
M3	0.689	20	M57	0.227	133	M111	0.288	109	M165	0.305	102
M4	0.428	52	M58	0.104	185	M112	0.359	77	M166	0.913	5
M5	0.259	118	M59	0.228	131	M113	0.233	127	M167	0.200	147
M6	0.104	186	M60	0.324	92	M114	0.414	59	M168	0.253	120
M7	0.257	119	M61	0.120	182	M115	0.496	37	M169	0.319	97
M8	0.157	169	M62	0.121	181	M116	0.843	9	M170	0.429	50
M9	0.342	86	M63	0.263	116	M117	0.057	194	M171	0.540	29
M10	0.537	30	M64	0.357	79	M118	0.239	123	M172	0.072	191
M11	0.186	153	M65	-0.044	211	M119	0.182	157	M173	0.351	82
M12	0.070	192	M66	0.332	88	M120	0.856	8	M174	0.149	172
M13	0.379	73	M67	0.980	4	M121	0.130	179	M175	0.458	43
M14	0.473	40	M68	0.212	141	M122	0.307	100	M176	0.214	140
M15	0.425	56	M69	0.222	138	M123	0.293	105	M177	0.302	103
M16	0.377	76	M70	0.216	139	M124	0.209	142	M178	0.081	188
M17	0.185	154	M71	0.045	197	M125	0.378	75	M179	0.138	174
M18	0.207	144	M72	0.429	48	M126	0.222	137	M180	0.016	203
M19	0.260	117	M73	-0.038	210	M127	0.327	90	M181	0.155	171
M20	0.169	162	M74	0.050	195	M128	0.246	122	M182	0.173	159
M21	0.416	57	M75	0.226	134	M129	0.428	51	M183	0.504	34
M22	0.622	25	M76	0.884	7	M130	-0.046	212	M184	0.177	158
M23	0.447	45	M77	0.708	16	M131	0.400	65	M185	0.352	81
M24	0.203	145	M78	0.036	199	M132	0.392	68	M186	0.384	72
M25	0.235	125	M79	0.049	196	M133	0.250	121	M187	0.182	156
M26	0.392	69	M80	0.064	193	M134	0.022	202	M188	0.076	190
M27	0.379	74	M81	0.235	126	M135	0.126	180	M189	0.737	15
M28	0.465	41	M82	-0.016	207	M136	0.338	87	M190	0.039	198
M29	-0.016	206	M83	0.132	177	M137	0.231	130	M191	0.134	176
M30	0.740	14	M84	-0.087	214	M138	0.393	67	M192	0.233	128
M31	0.386	71	M85	0.326	91	M139	0.224	135	M193	0.185	155
M32	0.359	78	M86	0.314	98	M140	0.405	62	M194	0.342	85
M33	0.388	70	M87	0.642	22	M141	-0.021	209	M195	0.319	96
M34	0.411	60	M88	0.291	106	M142	0.158	168	M196	0.686	21
M35	0.160	167	M89	0.356	80	M143	0.694	19	M197	0.628	24
M36	0.187	152	M90	0.425	55	M144	0.523	31	M198	0.190	150
M37	0.092	187	M91	0.273	114	M145	0.437	47	M199	0.775	12
M38	0.704	17	M92	0.227	132	M146	0.300	104	M200	0.765	13
M39	0.427	54	M93	0.188	151	M147	1.079	1	M201	0.222	136
M40	0.402	64	M94	0.452	44	M148	0.634	23	M202	0.265	115
M41	0.277	112	M95	0.164	164	M149	0.131	178	M203	0.190	149
M42	0.232	129	M96	0.136	175	M150	-0.017	208	M204	0.399	66
M43	0.277	111	M97	0.404	63	M151	0.109	184	M205	0.696	18
M44	0.415	58	M98	0.515	32	M152	0.156	170	M206	0.332	89
M45	0.276	113	M99	0.995	3	M153	0.146	173	M207	0.201	146
M46	0.460	42	M100	0.307	101	M154	0.802	10	M208	0.079	189
M47	0.008	204	M101	0.281	110	M155	0.349	83	M209	0.498	36
M48	0.289	108	M102	0.235	124	M156	0.429	49	M210	0.784	11
M49	0.162	165	M103	0.312	99	M157	0.503	35	M211	0.323	93
M50	0.169	161	M104	0.320	94	M158	0.562	28	M212	0.475	39
M51	0.348	84	M105	0.171	160	M159	0.475	38	M213	0.427	53
M52	0.110	183	M106	0.510	33	M160	0.405	61	M214	0.598	26
M53	0.208	143	M107	0.161	166	M161	0.999	2			
M54	0.164	163	M108	0.022	201	M162	-0.086	213			

Table S5. Grading range and strain distribution proportion of texture parameters of *Morchella* pileus.

Strain species	Characteristic	Grade	Traditional grading method		Least significant difference method		Probability grading method	
			Range	Distribution Proportion/%	Range	Distribution Proportion/%	Range	Distribution Proportion/%
M.importuna	Hardness	1	≤17.30	0.00	⩽38.69	3.28	⩽34.45	3.28
		2	17.31~44.90	8.20	38.70~66.29	62.30	34.45~51.08	22.95
		3	44.91~72.50	68.85	66.30~93.88	29.51	51.08~74.14	54.10
		4	72.51~100.10	18.03	93.89~121.48	3.28	74.14~90.77	14.75
		5	≥100.11	4.92	⩾124.49	1.64	⩾90.77	4.92
	Springiness	1	≤0.43	6.56	⩽0.38	1.64	⩽0.38	1.64
		2	0.44~0.67	86.89	0.39~0.52	37.70	0.38~0.48	21.31
		3	≥0.68	6.56	0.53~0.67	54.10	0.48~0.62	57.38
		4	—	—	0.68~0.81	6.56	0.62~0.72	18.03
		5	—	—	⩾0.82	0.00	⩾0.72	1.64
	Cohesiveness	1	≤0.27	18.03	⩽0.29	21.31	⩽0.31	26.23
		2	0.28~0.43	72.13	0.30~0.46	73.77	0.31~0.41	50.82
		3	≥0.44	9.84	⩾0.47	4.92	⩾0.41	22.95
	Gumminess	1	⩽10.46	1.64	⩽15.59	19.67	⩽8.61	1.64
		2	10.47~31.58	85.25	15.60~28.27	59.02	8.61~17.05	29.51
		3	⩾31.59	13.11	28.28~40.94	18.03	17.05~28.73	50.82
		4	—	—	40.95~53.62	3.28	28.73~37.17	11.48
		5	—	—	⩾53.63	0.00	⩾37.17	6.56
	Chewiness	1	⩽3.95	1.64	⩽16.65	72.13	⩽8.97	27.87
		2	3.96~19.43	81.97	16.66~32.14	27.87	8.97~17.73	50.82
		3	⩾19.44	16.39	⩾32.15	0.00	⩾17.73	21.31
	Resilience	1	⩽0.06	6.56	⩽0.05	3.28	⩽0.06	3.28
		2	0.07~0.15	80.33	0.06~0.11	59.02	0.06~0.09	29.51
		3	⩾0.16	13.11	0.12~0.18	32.79	0.09~0.15	47.54
		4	—	—	0.19~0.24	4.92	0.15~0.18	14.75
		5	—	—	⩾0.25	0.00	⩾0.18	4.92
M.sextelata	Hardness	1	≤40.91	9.09	⩽39.15	7.07	⩽34.80	1.01
		2	40.92~72.04	76.77	39.16~57.83	51.52	34.80~48.58	21.21
		3	≥72.05	14.14	57.84~76.52	30.30	48.58~67.66	57.58
		4	—	—	76.53~95.20	10.10	67.66~81.44	15.15
		5	—	—	⩾95.21	1.01	⩾81.44	5.05
	Springiness	1	≤0.38	10.10	⩽0.40	14.14	⩽0.44	25.25
		2	0.39~0.64	79.80	0.40~0.65	76.77	0.44~0.60	50.51
		3	≥0.65	10.10	⩾0.65	9.09	⩾0.60	24.24
	Cohesiveness	1	⩽0.18	7.07	⩽0.17	4.04	⩽0.16	1.01
		2	0.19~0.40	82.83	0.18~0.31	52.53	0.16~0.24	18.18
		3	⩾0.41	10.10	0.32~0.44	39.39	0.24~0.36	57.58
		4	—	—	0.45~0.58	4.04	0.36~0.44	16.16
		5	—	—	⩾0.59	0.00	⩾0.44	5.05
	Gumminess	1	⩽7.63	5.05	⩽13.12	24.24	⩽5.60	1.01
		2	7.64~25.73	67.68	13.13~23.98	57.58	5.60~13.17	25.25
		3	⩾25.74	17.17	23.99~34.85	14.14	13.17~23.67	54.55
		4	—	—	34.86~45.71	4.04	23.67~31.24	12.12
		5	—	—	⩾45.72	0.00	⩾31.24	7.07
	Chewiness	1	⩽2.32	2.02	⩽12.24	70.71	⩽6.45	26.26
		2	2.33~14.34	74.75	12.25~24.35	27.27	6.45~14.61	50.51
		3	⩾14.35	23.23	⩾24.36	2.02	⩾14.61	23.23
	Resilience	1	⩽0.03	7.07	⩽0.04	10.10	⩽0.03	3.03
		2	0.04~0.10	79.80	0.05~0.09	57.58	0.03~0.06	30.30
		3	⩾0.11	13.13	0.10~0.14	30.30	0.06~0.10	49.49
		4	—	—	0.15~0.19	2.02	0.10~0.13	11.11

		5	—	—	⩾0.20	0.00	⩾0.13	6.06
M.eximia	Hardness	1	≤47.73	7.41	⩽50.96	12.96	⩽40.15	1.85
		2	47.74~88.01	75.93	50.97~75.13	57.41	40.15~58.37	18.52
		3	≥88.02	16.67	75.14~99.29	18.52	58.37~83.61	61.11
		4	—	—	99.30~123.46	11.11	83.61~101.83	7.41
		5	—	—	⩾2123.47	0.00	⩾101.83	11.11
	Springiness	1	≤0.45	7.41	⩽0.35	0.00	⩽0.41	1.85
		2	0.46~0.69	81.48	0.36~0.50	18.52	0.41~0.51	24.07
		3	≥0.70	11.11	0.51~0.64	59.26	0.51~0.65	53.70
		4	—	—	0.65~0.79	18.52	0.65~0.75	18.52
		5	—	—	⩾0.80	1.85	⩾0.75	1.85
	Cohesiveness	1	≤0.27	5.56	⩽0.22	1.85	⩽0.24	3.70
		2	0.28~0.44	81.48	0.23~0.33	37.04	0.24~0.31	20.37
		3	≥0.45	12.96	0.34~0.43	48.15	0.31~0.41	59.26
		4	—	—	0.44~0.54	12.96	0.41~0.48	14.81
		5	—	—	⩾0.55	0.00	⩾0.48	1.85
	Gumminess	1	⩽13.49	5.56	⩽17.82	14.81	⩽10.66	1.85
		2	13.50~33.72	79.63	17.83~29.96	57.41	10.66~19.77	22.22
		3	⩾33.73	14.81	29.97~42.10	22.22	19.77~32.39	57.41
		4	—	—	42.11~54.24	3.70	32.39~41.50	12.96
		5	—	—	⩾54.25	1.85	⩾41.50	5.56
	Chewiness	1	⩽6.39	1.85	⩽10.46	24.07	⩽4.19	1.85
		2	6.40~20.44	77.78	10.47~18.89	51.85	4.19~10.96	25.93
		3	⩾20.45	20.37	18.90~27.33	20.37	10.96~20.32	53.70
		4	—	—	27.34~35.76	3.70	20.32~27.09	16.67
		5	—	—	⩾35.77	0.00	⩾27.09	3.70
	Resilience	1	⩽0.05	7.41	⩽0.06	12.96	⩽0.05	7.41
		2	0.06~0.13	72.22	0.07~0.11	61.11	0.05~0.08	27.78
		3	⩾0.14	20.37	0.12~0.17	20.37	0.08~0.14	50.00
		4	—	—	0.18~0.22	5.56	0.14~0.17	7.41
		5	—	—	⩾0.23	0.00	⩾0.17	5.56

Note: “—"indicates no data

Table S6. Grading range and strain distribution proportion of texture parameters of *Morchella* stipe.

Strain species	Characteristic	Grade	Traditional grading method		Least significant difference method		Probability grading method	
			Range	Distribution Proportion/%	Range	Distribution Proportion/%	Range	Distribution Proportion/%
M.importuna	Hardness	1	⩽72.00	4.92	⩽136.23	59.02	⩽104.72	34.43
		2	72.01~173.96	75.41	136.24~238.21	36.07	104.72~167.18	44.26
		3	⩾173.97	19.67	⩾238.22	4.92	⩾167.18	21.31
	Springiness	1	⩽0.64	13.11	⩽0.62	8.20	⩽0.67	21.31
		2	0.65~0.80	85.25	0.63~0.79	88.52	0.67~0.75	54.10
		3	⩾0.81	1.64	⩾0.80	3.28	⩾0.75	24.59
	Cohesiveness	1	≤0.56	13.11	⩽0.55	8.20	⩽0.60	27.87
		2	0.57~0.73	83.61	0.56~0.73	88.52	0.60~0.70	52.46
		3	≥0.74	6.56	⩾0.74	3.28	⩾0.70	19.67
	Gumminess	1	⩽52.64	8.20	⩽55.55	11.48	⩽45.22	4.92
		2	52.65~109.91	78.69	55.56~89.92	45.90	45.22~69.13	27.87
		3	⩾109.92	13.11	89.93~124.30	34.43	69.13~102.27	44.26
		4	—	—	124.31~158.67	6.56	102.27~126.18	14.75
		5	—	—	⩾158.68	1.64	⩾126.18	8.20
	Chewiness	1	≤11.83	0.00	⩽41.23	14.75	⩽30.23	4.92
		2	11.84~43.00	18.03	41.24~72.40	59.02	30.23~48.66	24.59
		3	43.01~74.17	60.66	72.41~103.58	22.95	48.66~74.18	49.18
		4	74.18~105.34	18.03	103.59~134.75	3.28	74.18~92.61	13.11

		5	≥105.35	3.28	≥134.76	0.00	≥92.61	8.20
		1	≤0.38	9.84	≤0.41	24.59	≤0.43	29.51
	Resilience	2	0.39~0.54	72.13	0.42~0.58	70.49	0.43~0.53	47.54
		3	≥0.55	18.03	≥0.59	4.92	≥0.53	22.95
		1	≤62.01	9.09	≤112.27	32.32	≤44.32	1.01
		2	62.02~200.31	78.79	112.28~195.26	53.54	44.32~102.71	27.27
	Hardness	3	≥200.32	12.12	195.27~278.24	8.08	102.71~183.61	54.55
		4	—	—	278.25~361.23	4.04	183.61~242.00	7.07
		5	—	—	≥361.24	2.02	≥242.00	10.10
		1	≤0.59	5.05	≤0.49	0.00	≤0.58	2.02
		2	0.60~0.75	86.87	0.50~0.59	6.06	0.58~0.64	25.25
	Springiness	3	≥0.76	8.08	0.60~0.69	59.60	0.64~0.72	53.54
		4	—	—	0.70~0.79	34.34	0.72~0.78	16.16
		5	—	—	≥0.80	0.00	≥0.78	3.03
		1	≤0.53	8.08	≤0.46	0.00	≤0.52	3.03
		2	0.54~0.70	82.83	0.47~0.57	27.27	0.52~0.58	25.25
	Cohesiveness	3	≥0.71	9.09	0.58~0.67	56.57	0.58~0.66	45.45
		4	—	—	0.68~0.78	16.16	0.66~0.72	20.20
M.sextelata		5	—	—	≥0.79	0.00	≥0.72	6.06
		1	≤45.05	13.13	≤64.43	28.28	≤33.09	4.04
		2	45.06~116.73	70.71	64.44~107.44	53.54	33.09~64.39	24.24
	Gumminess	3	≥116.74	16.16	107.45~150.45	12.12	64.39~107.77	53.54
		4	—	—	150.46~193.46	4.04	107.77~139.07	11.11
		5	—	—	≥193.47	2.02	≥139.07	7.07
		1	≤25.07	8.08	≤43.36	27.27	≤21.05	4.04
		2	25.08~81.82	74.75	43.37~77.42	54.55	21.05~43.20	22.22
	Chewiness	3	≥81.83	17.17	77.43~111.47	13.13	43.20~73.88	53.54
		4	—	—	111.48~145.53	5.05	73.88~96.03	13.13
		5	—	—	≥145.54	0.00	≥96.03	7.07
		1	≤0.30	0.00	≤0.31	0.00	≤0.36	3.03
		2	0.31~0.40	19.19	0.32~0.41	32.32	0.36~0.41	18.18
	Resilience	3	0.41~0.50	58.59	0.42~0.50	46.46	0.41~0.49	52.53
		4	0.51~0.60	21.21	0.51~0.60	20.20	0.49~0.54	20.20
		5	≥0.61	1.01	≥0.61	1.01	≥0.54	6.06
		1	≤78.12	9.26	≤89.82	18.52	≤51.26	3.70
		2	78.13~185.78	66.67	89.83~154.43	42.59	51.26~107.55	33.33
	Hardness	3	≥185.79	24.07	154.44~219.03	24.07	107.55~185.55	38.89
		4	—	—	219.04~283.64	9.26	185.55~241.84	14.81
		5	—	—	≥283.65	5.56	≥241.84	9.26
		1	≤0.63	5.56	≤0.56	0.00	≤0.61	1.85
		2	0.64~0.77	90.74	0.57~0.65	14.81	0.61~0.66	12.96
	Springiness	3	≥0.78	3.70	0.66~0.75	72.22	0.66~0.74	64.81
		4	—	—	0.76~0.84	12.96	0.74~0.79	18.52
		5	—	—	≥0.85	0.00	≥0.79	1.85
		1	≤0.53	3.70	≤0.46	0.00	≤0.50	1.85
M.eximia		2	0.54~0.69	75.93	0.47~0.56	24.07	0.50~0.57	22.22
	Cohesiveness	3	≥0.70	20.37	0.57~0.66	48.15	0.57~0.67	48.15
		4	—	—	0.67~0.76	27.78	0.67~0.74	25.93
		5	—	—	≥0.77	0.00	≥0.74	1.85
		1	≤51.18	9.26	≤52.19	9.26	≤38.22	3.70
		2	51.19~110.39	64.81	52.20~87.72	48.15	38.22~67.45	25.93
	Gumminess	3	≥110.40	25.93	87.73~123.25	25.93	67.45~107.95	44.44
		4	—	—	123.26~158.78	14.81	107.95~137.18	14.81
		5	—	—	≥158.79	1.85	≥137.18	11.11
		1	≤29.75	3.70	≤43.25	24.07	≤23.89	1.85
	Chewiness	2	29.76~84.47	75.93	43.26~76.08	51.85	23.89~46.43	24.07
		3	≥84.48	20.37	76.09~108.92	18.52	46.43~77.65	51.85
		4	—	—	108.93~141.75	5.56	77.65~100.19	9.26

	5	—	—	⩾141.76	0.00	⩾100.19	12.96
Resilience	1	≤0.23	0.00	⩽0.36	5.56	⩽0.33	1.85
	2	0.24~0.35	3.70	0.37~0.48	85.19	0.33~0.39	31.48
	3	0.36~0.47	75.93	0.49~0.61	7.41	0.39~0.47	42.59
	4	0.48~0.59	18.52	0.62~0.73	1.85	0.47~0.53	18.52
	5	≥0.60	1.85	⩾0.74	0.00	⩾0.53	5.56

Note: footnotes as in Table S5
